# Changes in Functional Brain Connectivity Highlight the Importance of a Baseline Measurement and Multiple Imaging Sessions for Cocaine Abstinence Studies

**DOI:** 10.1523/ENEURO.0372-18.2018

**Published:** 2018-10-02

**Authors:** Rosalind S.E. Carney

## Abstract

**Highlighted Research Paper:** Functional Connectivity of Chronic Cocaine Use Reveals Progressive Neuroadaptations in Neocortical, Striatal, and Limbic Networks by, Caitlin A. Orsini, Luis M. Colon-Perez, Sara C. Heshmati, Barry Setlow, and Marcelo Febo

Human neuroimaging studies have shown that chronic cocaine use leads to short- and long-term defects in brain function. In the short term, synaptic dopamine levels and glutamate transmission are altered (Kalivas et al., 2009; Volkow and Morales, 2015). In the long term, a reduction in neural activity occurs between certain brain regions, such as the prefrontal cortex (PFC), amygdala, and hippocampus (Gu et al., 2010). Abstinent cocaine users show reduced interhemispheric functional connectivity in the lateral PFC, medial premotor, and lateral parietal cortices (Kelly et al., 2011), whereas relapsed cocaine users exhibit reduced connectivity between the corticomedial amygdala and ventromedial and rostral anterior cingulate cortices (McHugh et al., 2014). Although such studies in humans generate important insights, their cross-sectional nature and comparison to other individuals as controls precludes measuring baseline brain connectivity before cocaine use. Also, studies on human cocaine users may have other caveats, such as polydrug use, incorrect recall of drug use or amount, differences in duration of use, relapse, or abstinence. Such variations may confound some interpretations about the effects of cocaine on brain structures. Also, as no baseline data were obtained, there is a possibility that aberrant connectivity patterns do not result from cocaine use, rather, they could represent an earlier neurodevelopmental defect that contributed to the onset of the maladaptive behavior. As it is unlikely that human studies will include a baseline measurement before cocaine exposure, it is useful to study animal models to determine the adverse effects of cocaine use on brain structure. However, only one previous publication described changes in functional connectivity between a baseline measurement and one month of abstinence following cocaine exposure (Lu et al., 2014).

In their *eNeuro* publication, Orsini et al., 2018 compared changes in functional connectivity in the rat brain between a baseline measurement and two time points of abstinence following cocaine exposure. Three resting-state functional magnetic resonance imaging (fMRI) sessions (Fig. 1*A*) were conducted as follows.

**Figure 1.**
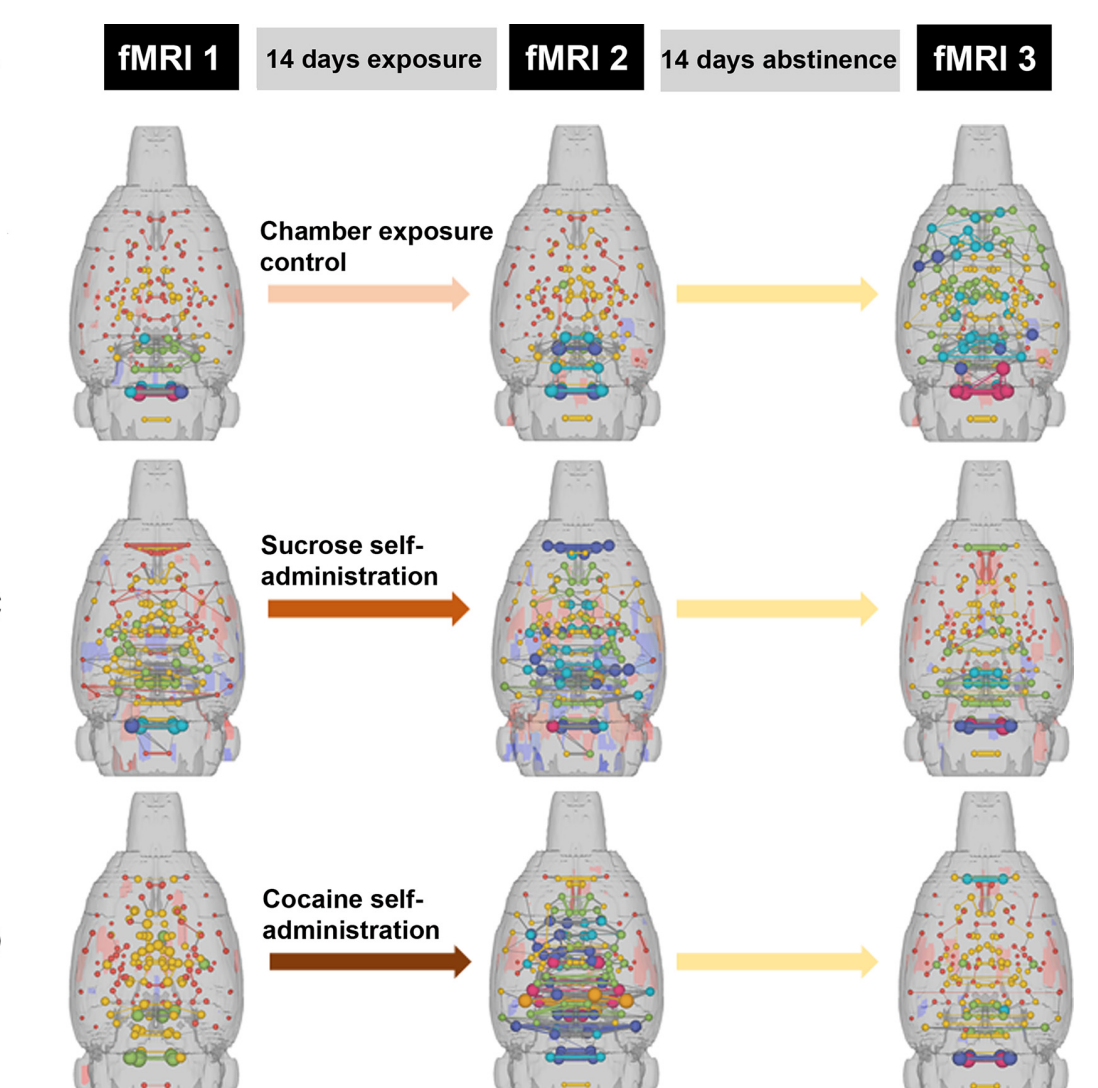
3D functional connectivity maps of the rat brain illustrating significant effects of sucrose or cocaine self-administration and abstinence. ***A***, Timeline of fMRIs with respect to exposure and abstinence periods. ***B–D***, 3D connectomic maps showing qualitative changes in functional connectivity during imaging sessions in response to chamber exposure, a control condition (***B***), sucrose self-administration (***C***), or cocaine self-administration (***D***). Spheres represent node strength and line thicknesses represent edge weights. (Adapted from Figures 1 and 4 in Orsini et al., 2018.)

## fMRI 1

Represents the baseline measurement. All rats were subsequently implanted with a catheter in the jugular vein and then divided into three groups. One group was trained in 6-h sessions in operant chambers to make a nose poke response to obtain intravenous cocaine. A second group was trained to make the same response to obtain access to a liquid sucrose reward. Rats in this second group were “yoked” to partner rats in the cocaine group such that they were restricted to earning the same number of sucrose rewards as their partner rats had earned cocaine rewards. The third group was placed in the operant chambers but received no training procedures. All groups were exposed to their respective conditions for 14 d.

## fMR1 2

Performed 1 d after exposure period ended. This represents day 1 of abstinence (1d Abs).

## fMR1 3

Performed at day 14 of abstinence (14d Abs), a time point at which previous studies have demonstrated neurobiological or behavioral changes relative to 1d Abs (Doyle et al., 2014; Glynn et al., 2018).

Orsini and colleagues performed several types of analyses of the fMRI data to assess functional connectivity, i.e., structures (nodes) that are associated by vascular, hemodynamic, and somatodendritic activity. In this context, functional connectivity does not necessarily imply direct axonal connectivity but can include structures that are associated by neural activity.

Figure 1 shows 3D connectomic maps created from each fMRI imaging session demonstrating node strength (spheres) and edge weights (lines connecting spheres) in the chamber exposure control group (Fig. 1*B*), the sucrose self-administration group (Fig. 1*C*), and the cocaine self-administration group (Fig. 1*D*). Chamber exposure controls showed no difference between baseline and 1d Abs yet exhibited an increase in cortical functional connectivity at 14d Abs. Sucrose self-administration resulted in increased connectivity in subcortical areas at 1d Abs. Cocaine self-administration led to increased functional connectivity in subcortical areas, including thalamic, hypothalamic, and forebrain regions at 1d Abs, whereas decreased functional connectivity was observed at 14d Abs. Although these observations are qualitative in nature and do not identify nodes to a precise anatomic level, these data show that changes in brain function can occur quite rapidly within a single period of abstinence. Most importantly, the data also highlight the important of a baseline measurement in substance-exposure studies.

Orsini and colleagues did not observe the same cocaine-induced changes in functional activity as detailed in the study by Lu et al. (2014). However, this could be explained by differences in methodology. As Lu et al. (2014) performed the postexposure fMRI at one month of abstinence, it is possible that the changes that they observed had not yet manifest in the cocaine-exposed rats analyzed at 14d Abs by Orsini and colleagues. This discrepancy supports the use of multiple postexposure time points of fMRI imaging in future studies to capture as many functional alterations in brain connectivity as possible.

Assistant Professor Marcelo Febo and Professor Barry Setlow (both at the University of Florida) are pursuing this line of research further to determine the molecular changes that occur as a result of cocaine exposure. For example, correlating changes in functional connectivity detected by fMRI with dopamine receptor expression. Also, the fMRI observations could be related to behavioral modifications, such as decision making or impulsivity. Other directions could include other drugs of abuse or other imaging methods such as diffusion MRI to identify major tracts in the brain. And importantly, what are methods of restoration of functional connectivity following substance abuse, which could be examined using optogenetics or compounds that may reduce addictive behaviors.

## References

[B1] Doyle SE, Ramôa C, Garber G, Newman J, Toor Z, Lynch WJ (2014) A shift in the role of glutamatergic signaling in the nucleus accumbens core with the development of an addicted phenotype. Biol Psychiatry 76:810–815. 10.1016/j.biopsych.2014.02.005 24629536 PMC4133320

[B2] Glynn RM, Rosenkranz JA, Wolf ME, Caccamise A, Shroff F, Smith AB, Loweth JA (2018) Repeated restraint stress exposure during early withdrawal accelerates incubation of cue-induced cocaine craving. Addict Biol 23:80–89. 10.1111/adb.12475 27859963 PMC5426993

[B3] Gu H, Salmeron BJ, Ross TJ, Geng X, Zhan W, Stein EA, Yang Y (2010) Mesocorticolimbic circuits are impaired in chronic cocaine users as demonstrated by resting-state functional connectivity. Neuroimage 53:593–601. 10.1016/j.neuroimage.2010.06.066 20603217 PMC2930044

[B4] Kalivas PW, Lalumiere RT, Knackstedt L, Shen H (2009) Glutamate transmission in addiction. Neuropharmacology 56 [Suppl 1]:169–173. 10.1016/j.neuropharm.2008.07.011 18675832 PMC3280337

[B5] Kelly C, Zuo XN, Gotimer K, Cox CL, Lynch L, Brock D, Imperati D, Garavan H, Rotrosen J, Castellanos FX, Milham MP (2011) Reduced interhemispheric resting state functional connectivity in cocaine addiction. Biol Psychiatry 69:684–692. 10.1016/j.biopsych.2010.11.022 21251646 PMC3056937

[B6] Lu H, Zou Q, Chefer S, Ross TJ, Vaupel DB, Guillem K, Rea WP, Yang Y, Peoples LL, Stein EA (2014) Abstinence from cocaine and sucrose self-administration reveals altered mesocorticolimbic circuit connectivity by resting state MRI. Brain Connect 4:499–510. 10.1089/brain.2014.0264 24999822 PMC4146381

[B7] McHugh MJ, Demers CH, Salmeron BJ, Devous MD Sr, Stein EA, Adinoff B (2014) Cortico-amygdala coupling as a marker of early relapse risk in cocaine-addicted individuals. Front Psychiatry 5:16. 10.3389/fpsyt.2014.00016 24578695 PMC3936467

[B9] Orsini CA, Colon-Perez LM, Heshmati SC, Setlow B, Febo M (2018) Functional Connectivity of Chronic Cocaine Use Reveals Progressive Neuroadaptations in Neocortical, Striatal, and Limbic Network eNeuro 4:ENEURO.0081-18.2018. CrossRef 10.1523/ENEURO.0081-18.2018 PMC6071197 30073194

[B8] Volkow ND, Morales M (2015) The brain on drugs: from reward to addiction. Cell 162:712–725. 10.1016/j.cell.2015.07.046 26276628

